# Does the absence of the load effect imply the absence of the dilution effect?

**DOI:** 10.3389/fcogn.2025.1558164

**Published:** 2025-09-05

**Authors:** Hanna Benoni

**Affiliations:** Department of Psychology, The College of Management, Academic Studies, Rishon LeZion, Israel

**Keywords:** perceptual load, dilution, selective attention, dilution vs. perceptual load, reversed load effect, distractor interference

## Introduction

A central goal of selective attention research is to determine under what conditions individuals can effectively focus on task-relevant targets while filtering out distractors. One influential line of research has shown that, counterintuitively, increasing the number of irrelevant neutral stimuli in the visual field reduces distractor interference. This set size effect has traditionally been interpreted within the framework of Perceptual Load Theory (Lavie, 1995; Lavie and Tsal, 1994), which attributes the effect to increased task difficulty associated with processing a larger number of irrelevant items, leaving insufficient capacity for processing distractors. The set size effect has also been explained by an alternative account—the Dilution account (Benoni and Tsal, 2010; Tsal and Benoni, 2010; Wilson et al., 2011), which attributes the effect to representational competition between the distractor and additional irrelevant stimuli, irrespective of task load.

Several recent studies have drawn conclusions about the dilution account based solely on manipulations of perceptual load, without directly testing dilution through a specific condition. This Opinion article offers a focused commentary on this recurring methodological, interpretational, and conceptual issue. The structure of the article is as follows: first, I briefly introduce the perceptual load theory and the dilution account, highlighting the conceptual distinction between them. Next, I present a representative review of studies that have inferred conclusions about the dilution account based solely on the manipulation of perceptual load. I then outline the conceptual, methodological, and inferential flaws in this approach and support the critique with empirical findings. Finally, I offer a summary and propose directions for future research.

## A brief introduction to perceptual load theory and the dilution account

*Perceptual Load Theory (PLT)* (Lavie, 1995; Lavie and Tsal, 1994; Lavie, 2005), one of the most influential theories of attention, proposes that the processing load of the relevant task determines the extent to which irrelevant distractors are processed. If target processing does not exhaust attentional resources (i.e., under low task load), leftover resources will spill over to process distractors, thereby producing interference. However, when target processing depletes all attentional resources (under high task load), distractor processing can be prevented. In a classic set-size manipulation of perceptual load, participants are required to identify a target letter that appears either alone (Low-Load condition) or among non-target neutral letters (High-Load condition). The efficiency of selection is measured by the effect of an incongruent vs. a congruent distractor appearing in the periphery (Flanker Effect). Typically, consistent with PLT predictions, substantial distractor interference under Low-Load conditions is reduced or eliminated under High-Load displays (e.g., Lavie and Cox, 1997; Maylor and Lavie, 1998; Lavie and Fox, 2000; Forster and Lavie, 2007). This beneficial effect of high set-size displays on selection has been interpreted as the *Perceptual Load Effect (PLE)*, assuming that task load, manipulated via display set size, causes this effect.

*The Dilution account* (Benoni and Tsal, 2010; Tsal and Benoni, 2010; Wilson et al., 2011) was proposed as an alternative framework for interpreting the so-called PLE and understanding how high set-size displays influence distractor processing. It suggests that the reduction in distractor interference under High-Load conditions need not be attributed to increased task demands, namely, the effort required to search for the target among neutral items, as proposed by PLT—but may instead result from dilution: the weakening of distractor processing due to the presence of additional neutral stimuli. These stimuli may compete with the distractor, irrespective of task load.

**Figure 1:**
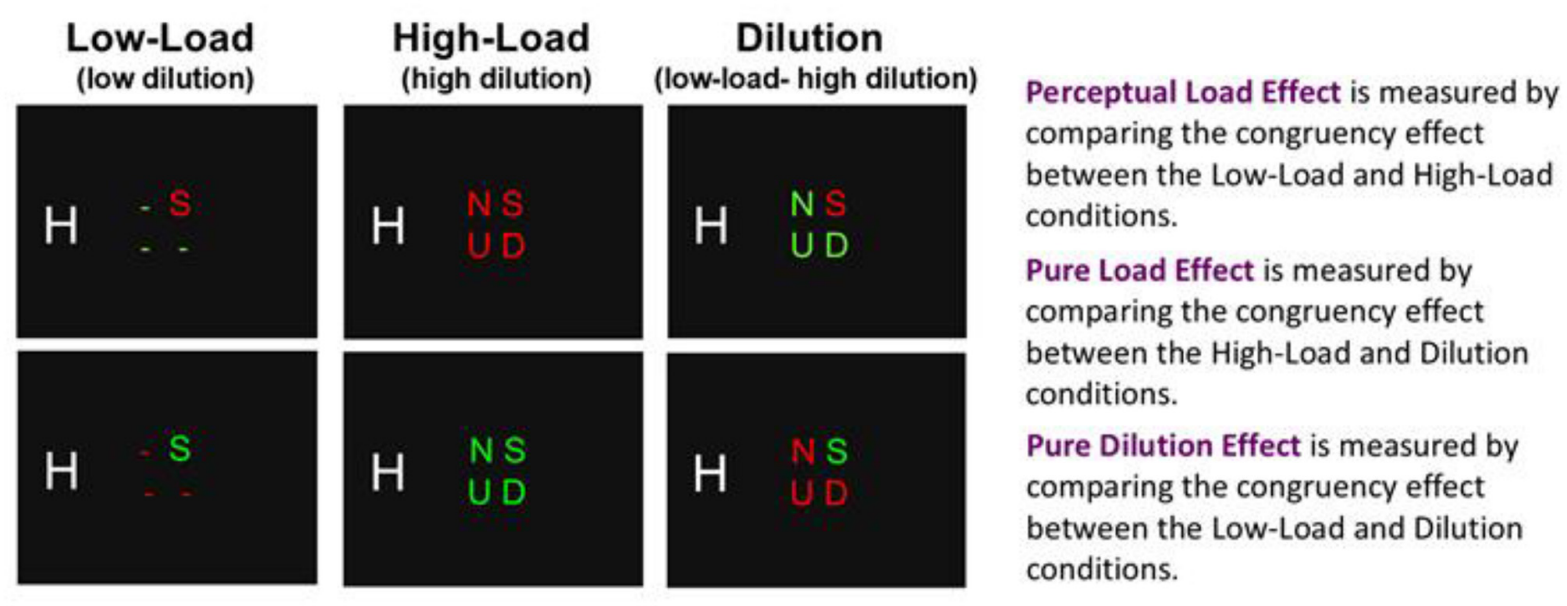
Examples of stimulus displays from Experiment 1 of Benoni and Tsal (2010), representing three experimental conditions: low-Load (low dilution), High-Load (high dilution), and a “Dilution” condition (low-load with high dilution). The illustrated displays depict incongruent trials, in which the distractor letter (“H”) differs from the target letter (“S”). In congruent trials, both the distractor and the target share the same identity (e.g., both are “S” or both are “H”).

To test this hypothesis, Benoni and Tsal (2010), Tsal and Benoni (2010), and Wilson et al. (2011) introduced a critical condition characterized by low perceptual load but high dilution potential. These dilution displays included neutral letters, similar to High-Load displays, but were designed to minimize task demands by eliminating the need to search for the target. For example, in this condition, the target differed in color from the heterogeneous neutral letters, making it easily distinguishable and enabling Low-Load processing despite the presence of multiple items (e.g., Benoni and Tsal, 2010, Experiment 1). Figure 1 illustrates the distinction between load and dilution effects based on these displays.

Across the experiments in these studies, which used varied converging operations, distractor interference in the Dilution condition was either eliminated or markedly reduced compared to the original Low-Load condition. These findings support the view that the reduction of interference under High-Load conditions, typically attributed to load, may, in fact, result from dilution (see also Benoni and Tsal, 2012; Benoni et al., 2014; Dittrich and Stahl, 2011; Marciano and Yeshurun, 2011; Mevorach et al., 2014; Tan et al., 2018).

## Studies challenging the dilution account based on null load effects: a representative review

Several studies have questioned Perceptual Load Theory (PLT) independently of the dilution account, based on alternative considerations. These studies manipulated variables that influence attentional deployment or post-perceptual processes, and have shown that distractor interference can remain significant, or even increase, under High-Load conditions compared to Low-Load conditions (e.g., Biggs and Gibson, 2010; Eltiti et al., 2005; Lleras et al., 2017; Marciano and Yeshurun, 2011; Normand et al., 2015; Theeuwes et al., 2004; Roper and Vecera, 2013; Yeshurun and Marciano, 2013).

Among these studies that question PLT due to the absence of a load effect, several have also challenged the dilution account based on the same observation (e.g., Cosman et al., 2016; Lleras et al., 2017; Eayrs et al., 2024; Weissman et al., 2018). The underlying reasoning appears to be as follows: if dilution is the mechanism responsible for the reduction in distractor interference observed in High-Load conditions, then such reduction should consistently occur whenever High-Load conditions are used. Therefore, the absence of reduced interference in these conditions is taken as evidence against the dilution mechanism.

For instance, in a recent study by Eayrs et al. (2024), participants completed a visual search task with intermixed High-, Low-, and Intermediate-Load trials, each preceded by a cue indicating the expected load level. The findings revealed that the reduction in distractor interference under High-Load was not consistent; it emerged only when participants both anticipated a High-Load trial and had previously experienced one. The authors argued that these results challenge not only perceptual load theory but also the dilution account, noting that “although the PLT and dilution accounts differ in the underlying mechanism of the effect, they both attribute reduced flanker interference in high load to the perceptual elements of the display” (p. 770).

In another study by Lleras et al. (2017), the authors revisited Forster and Lavie's (2008) procedure to test whether task load affects the susceptibility of the attention system to capture by entirely irrelevant stimuli. In this paradigm, as in typical load manipulations (Lavie and Cox, 1997), participants were tasked with identifying a target letter under Low- and High-Load conditions. Distractibility was assessed by comparing trials in which an irrelevant peripheral distractor (e.g., a real-world object or cartoon) appeared with non-distractor trials. While Lleras et al. replicated the perceptual load effect (PLE) under identical conditions to the original experiments, they observed that variations in the paradigm, such as increasing the proportion of distractor trials from 20 to 33% (Experiment 1) or extending the presentation time from 100 to 200 ms (Experiment 2), resulted in enhanced rather than reduced distractor interference under High-Load condition. The authors concluded that these findings challenge both the dilution account and the perceptual load theory, stating that “the two theories both predict reduced effects of the ‘irrelevant' stimulus on target processing in high perceptual load conditions” (p. 174).

## Does the absence of a load effect preclude a dilution effect?

Can the absence of a load effect truly inform us about the absence of a dilution effect? I argue that it cannot. The absence of a perceptual load effect does not constitute sufficient evidence against the existence of a dilution effect. Observations of the *reverse load effect*, as well as findings demonstrating dilution effects in the absence of perceptual load effects, suggest that valid conclusions about dilution require the inclusion of a dedicated dilution condition in the experimental design.

### The “reverse load effect”

Inference about the absence of a dilution effect from the absence of a perceptual load effect (PLE) is often based on an implicit assumption drawn from the early dilution studies (Benoni and Tsal, 2010; Tsal and Benoni, 2010), that the PLE is, in fact, a dilution effect. That is, that a single mechanism, namely dilution, accounts for the difference in distractor interference between Low- and High-Load displays. However, *a posteriori* findings from those early studies and subsequent work suggest otherwise. Specifically, comparisons between High-Load and Dilution conditions, originally assumed to produce similar levels of distractor interference, revealed an unexpected pattern in several manipulations: distractor interference was greater in the High-Load condition than in the Dilution condition (e.g., Tsal and Benoni, 2010; Benoni and Tsal, 2012; Wilson et al., 2011). This result, in which High-Load increases distractor interference relative to a condition that includes dilution but no search demands, has been termed the *reverse load effect*.

The reverse load effect suggests several important conclusions: First, it indicates that High-Load and Dilution conditions are not functionally equivalent with respect to distractor interference. Second, when considering the structure of the High-Load condition in set-size manipulations, it becomes evident that it consists of two components relative to the Low-Load condition: (1) increased task demands due to the need to search for the target among neutral items—the *load* component; and (2) the presence of additional neutral stimuli—the *dilution* component. Third, given that High-Load displays sometimes lead to *increased* distractor interference compared to Dilution displays, it follows that the *load* component may actually enhance distractor interference, working in the opposite direction of the dilution mechanism. This unexpected increase in distractor interference under High-Load conditions may have multiple interpretations. One possibility is that such displays impose cognitive demands—such as increased working memory load or reduced top-down control, thereby increasing distractor interference. While this explanation relies on cognitive rather than perceptual load mechanisms (Lavie, 2005), it further underscores the conceptual need to distinguish between dilution and load.

Taken together, these observations demonstrate that one cannot infer the absence of a dilution effect from the absence of a perceptual load effect. The two mechanisms may operate independently, and in some cases, even antagonistically.

### Data supporting dilution effects in the absence of perceptual load effects

Support for the idea of distinct 'load' and 'dilution' components was also obtained from the study by Benoni et al. (2014). Following the study by Theeuwes et al. (2004), the purpose of the study was to assess the potential separate effects of the viewers' attentional set (operationally manipulated through blocked vs. mixed designs) on load and dilution effects. This objective was achieved by replicating Theeuwes' load manipulation study, with the addition of a new Dilution condition. The results indicated that while attentional set interacted with task load (as shown in Theeuwes' study), it did not interact with “dilution.” Specifically, the Dilution condition reduced distractor interference compared to the Low-Load condition, in both mixed and fixed designs. These findings suggest that the viewers' attentional window influences the “load” component but not the “dilution” component. Furthermore, they may imply that dilution operates as a more automatic process, that is less affected by attentional allocation (see also Mevorach et al., 2014).

## Discussion

Evidence of the “reverse load effect” and data showing dilution effects in the absence of perceptual load effects suggest that the “dilution” component and the “load” component may independently influence distractor processing. Furthermore, it is possible that all attentional or post-perceptual factors shown to modulate PLEs in previous studies (e.g., Biggs and Gibson, 2010; Cosman et al., 2016; Eayrs et al., 2024; Eltiti et al., 2005; Lleras et al., 2017; Marciano and Yeshurun, 2011; Normand et al., 2015; Theeuwes et al., 2004; Roper and Vecera, 2013; Yeshurun and Marciano, 2013) influence the “load” component but not the “dilution” component, or perhaps affect these components differently. The “load” component may confer certain advantages and disadvantages that are unrelated to the dilution mechanism. For instance, considering the idea that narrowing the attentional zoom is more efficient in High-Load conditions than in Low-Load conditions (e.g., Theeuwes et al., 2004), this advantage may result from active searching- that is, from “task load”- rather than from “dilution.” Similarly, the search function may lead to disadvantages, such as increased reaction time and, consequently, a higher probability of distraction, which are not inherent to the dilution component. Therefore, to draw valid conclusions about the influence of specific factors on dilution effects, it is crucial that research designs include a distinct Dilution condition.

Finally, distinguishing properly between dilution and task load is also important for understanding how these components may interact with alternative accounts proposed in the literature, such as attentional control settings (Theeuwes et al., 2004), relative activation (Yeh and Lin, 2013), and distractor saliency (Biggs and Gibson, 2014; see also Murphy et al., 2016, for a broader perspective and review of such alternatives). Methodologically, any attempt to assess the separate effects of dilution, load, and other variables must ensure that the dilution component is properly disentangled from task-related processing demands.
